# Targeting behavioral factors with digital health and shared decision-making to promote cardiac rehabilitation—a narrative review

**DOI:** 10.3389/fdgth.2024.1324544

**Published:** 2024-02-23

**Authors:** Isabel Höppchen, Daniela Wurhofer, Alexander Meschtscherjakov, Jan David Smeddinck, Stefan Tino Kulnik

**Affiliations:** ^1^ Ludwig Boltzmann Institute for Digital Health and Prevention, Salzburg, Austria; ^2^ Department of Artificial Intelligence and Human Interfaces, Human Computer Interaction Division, Paris Lodron University of Salzburg, Salzburg, Austria

**Keywords:** secondary prevention, behavior change, patient transition, cardiovascular disease, patient-centered

## Abstract

Cardiac rehabilitation (CR) represents an important steppingstone for many cardiac patients into a more heart-healthy lifestyle to prevent premature death and improve quality of life years. However, CR is underutilized worldwide. In order to support the development of targeted digital health interventions, this narrative review (I) provides understandings of factors influencing CR utilization from a behavioral perspective, (II) discusses the potential of digital health technologies (DHTs) to address barriers and reinforce facilitators to CR, and (III) outlines how DHTs could incorporate shared decision-making to support CR utilization. A narrative search of reviews in Web of Science and PubMed was conducted to summarize evidence on factors influencing CR utilization. The factors were grouped according to the *Behaviour Change Wheel*. Patients' *Capability* for participating in CR is influenced by their disease knowledge, awareness of the benefits of CR, information received, and interactions with healthcare professionals (HCP). The *Opportunity* to attend CR is impacted by healthcare system factors such as referral processes and HCPs' awareness, as well as personal resources including logistical challenges and comorbidities. Patients' *Motivation* to engage in CR is affected by emotions, factors such as gender, age, self-perception of fitness and control over the cardiac condition, as well as peer comparisons. Based on behavioral factors, this review identified intervention functions that could support an increase of CR uptake: Future DHTs aiming to support CR utilization may benefit from incorporating information for patients and HCP education, enabling disease management and collaboration along the patient pathway, and enhancing social support from relatives and peers. To conclude, considerations are made how future innovations could incorporate such functions.

## 1 Introduction

Cardiovascular diseases (CVDs) are a group of disorders of the heart and blood vessels, commonly causing heart attacks or strokes. CVD are the leading cause of death and are responsible for approximately 32% of all deaths globally (1). They are primarily caused by behavioral risk factors, for example, unhealthy diet, tobacco use, obesity, physical inactivity, and harmful use of alcohol (1). In addition to surgery and medication, cardiac rehabilitation (CR) is crucial for the secondary prevention of CVDs, i.e., preventing the occurrence of further acute cardiovascular events, reducing the risk of premature death and improving health-related quality of life. With its multi-faceted program centered around supervised exercise therapy, CR represents a steppingstone for many patients into a more heart-healthy lifestyle. Moreover, patients attend educational sessions, learning about blood pressure management, lipid and glycemic targets, heart-healthy nutrition, and tobacco cessation, and they receive psychological support (2). Throughout their secondary prevention pathway, patients' self-management capabilities are of great importance.

The positive effects of CR, namely a better heart health and function, less need for medication, the adoption of healthy behaviors, and a lower risk of cardiac mortality have been multiply confirmed (3). Therefore, the American Heart Association and the European Society for Cardiology recommend CR with the highest classification possible (4, 5). Nevertheless, evidence shows that CR is underutilized worldwide (6). The term *CR utilization* comprises four aspects (7). Firstly, the patient's referral to CR, which is usually conducted in hospitals. Secondly, the patient's enrolment in the CR program. Thirdly, the adherence rate as indicated by the proportion of sessions completed out of those prescribed. Finally, the reassessment after the CR intervention after program completion (7).

Previous research has shown that the reasons for CR underutilization comprise of an interplay of barriers addressing different stages of the patient pathway (6). Patient information and communication between healthcare professionals (HCPs) and patients were identified as fundamental factors for patients' acceptance of medical advice (8). For example, HCPs recommendation to participate in CR positively influences patients' motivation to participate. Although the barriers to CR have been thoroughly investigated, with the first publications dating back to 1992 (9), there is a lack of standardized reporting.

Frameworks such as the *Behaviour Change Wheel* (BCW) (10) can provide structure, guidance, and a systematic approach for developing and implementing (digital) interventions. Moreover, such frameworks help to deconstruct complex healthcare system-related challenges and support researchers and developers in creating targeted solutions. In cardiac care, frameworks focusing on behavioral factors have the potential to support the understanding of barriers and facilitators to patients' CR pathways. They could serve as a roadmap considering stakeholders' preferences, underlying needs, and social context. The insights could then build the basis for developing digital interventions reinforcing a targeted behavior, for example, the uptake of CR.

### 1.1 Shared decision-making on the patients' pathway to CR

Shared decision-making (SDM) is a collaborative decision process incorporating current medical evidence and patients’ personal preferences regarding their medical treatment. The approach focuses on patient-centered care and ethical perceptions of individuals' self-determination. Patients are considered stakeholders who are actively involved in the medical decision process while HCPs educate them about their options in layperson's terms. Treatment decisions are made jointly between patients and HCPs. Therefore, a trusting interpersonal relationship between HCPs and patients is necessary (11, 12).

Related work describes medical SDM in multiple models and frameworks (12). For example, Elwyn et al. (11) provide a three-step model as guidance on how SDM can be accomplished in routine clinical care: At first, a *choice talk* represents a planning step and aims to make the patients aware that reasonable treatment options exist. HCPs emphasize the importance of respecting preferences and inform the patients about making a decision. By checking patients' reactions, HCPs elicit to what extent patients want to be involved in the decision-making process. Next, HCPs list options including their harms and benefits according to patients' knowledge base. This *option talk* aims to provide decision support. Finally, a *decision talk* clarifies patients' questions and preferences, and moves towards a decision. HCPs close the discussion by offering to review the decision. The described steps can be iterated as often as necessary to ensure patients are clear about the options and can articulate their preferences and needs.

In cardiac care, related work indicates that patients' values and preferences for decision-making might change along their care pathway. Burton et al. (13) researched patients undergoing elective cardiac surgery and found that only 40% wanted to be involved in their treatment decisions. However, they also found that perceived involvement in decisions led to higher confidence regarding the decision (13). This finding aligns with evidence showing that cardiac patients who participate in SDM have a better understanding of the risks and benefits of treatment options (14). Bente et al. (15) investigated values of CVD patients facing lifestyle and behavior change. They found that patients wanted to be involved in decision making and expressed interest to oversee their health and treatment progress. Patients also preferred personalized care, considering their individual needs and preferences (15).

A structured SDM approach, including personalized patient education, may enhance awareness among patients about the option of CR. It can also address individual considerations influencing patients' decision to participate in CR. Enhancing patients' awareness and understanding of their condition and the role of CR can contribute to informed decisions regarding CR participation.

### 1.2 Digital health technologies supporting CR utilization

Digital health technologies (DHTs) can support the use of CR programs, e.g., by facilitating care processes and increasing patients' understanding of their condition. Technologies could also engage patients as proactive stakeholders beyond their time with HCPs. This includes educating them about treatment options and facilitating SDM.

In order to enhance CR uptake on the healthcare system level, related work describes automated rehabilitation referrals based on data from electronic medical records (16–19). However, such solutions fall short when it comes to considering patients on an individual level. As hospitalization time decreases due to highly condensed workflows and workforce shortages, the time for discussing follow-up care and educating patients about secondary prevention in the acute setting is limited (20). At this point, digital decision aids could take effect (21, 22). Through imparting knowledge and eliciting medical treatment options, they have the potential to enable and prepare patients for SDM with the HCPs. On the continuing care pathway, text message reminders and activity monitoring may support cardiac medication and rehabilitation adherence (23, 24).

Despite the promising potential of DHTs in supporting cardiac patients, they face criticism. One significant drawback is the limited quality of health technologies, which hinders their full impact in practice. Decision aids, for example, might be easily accessible online. However, their overall content quality is criticized as low, and some aids may not be suitable for groups with low literacy (25). This limitation can undermine the efficacy of supporting SDM, creating a potential digital divide in access to crucial information and guidance. Moreover, HCPs' concerns regarding the effectiveness and perceived workload associated with digital technologies hamper their implementation in practice (26). Consequently, the long-term evaluation of DHTs' effectiveness in improving patient outcomes and CR utilization remains an ongoing challenge. Research gaps persist in understanding the impact of DHTs on patient engagement, behavior change, and long-term health outcomes. The healthcare landscape is dynamic, and the rapid evolution of digital technologies introduces new challenges and opportunities that require continuous evaluation and adaptation. Technologies supporting medication adherence and rehabilitation show promise in the shorter term. More research is needed to assess their long-term effectiveness and ability to promote sustained behavioral change in patients.

## 2 Objectives

This narrative review aims to deepen the understanding of factors influencing CR utilization and the role of SDM in potential digital solutions. Using the BCW (10) as an underlying theory, we map factors influencing CR utilization to the behavior domains. We also highlight intervention functions for future health innovations designed for increasing CR utilization. We discuss how available DHTs already incorporate such functions to address barriers and reinforce facilitators to CR. We also outline how future DHTs might be designed and the role of SDM in this context.

In summary, our review (I) provides understandings of factors influencing CR utilization from a behavioral perspective, (II) discusses the potential of DHTs as solutions to address barriers and reinforce facilitators to CR uptake and (III) outlines how DHTs incorporate SDM to support CR utilization.

## 3 Methods

A narrative review was chosen as it allows a reflective analysis of the current evidence about factors influencing CR utilization. It also emphasizes the interpretation and the proposal of new ideas and concepts (27), and we make use of this by speculating on future healthcare innovations.

This review follows the *Scale for the Assessment of Narrative Review Articles* (28) to support research integrity and improve the standard of non-systematic reviews. It also follows the hermeneutic approach for literature reviews (29).

### 3.1 Literature search and inclusion process

In order to identify literature describing barriers and facilitators for CR, German and English review articles were searched in PubMed and Web of Science (Core Collection) databases. *Barrier*, *cardiac rehabilitation*, and *utilization* were defined as keywords. Keywords were combined in search strings with synonyms and Boolean operators for each database. An additional keyword search was conducted in Google Scholar (Supplementary Material 1).

**Figure 1:**
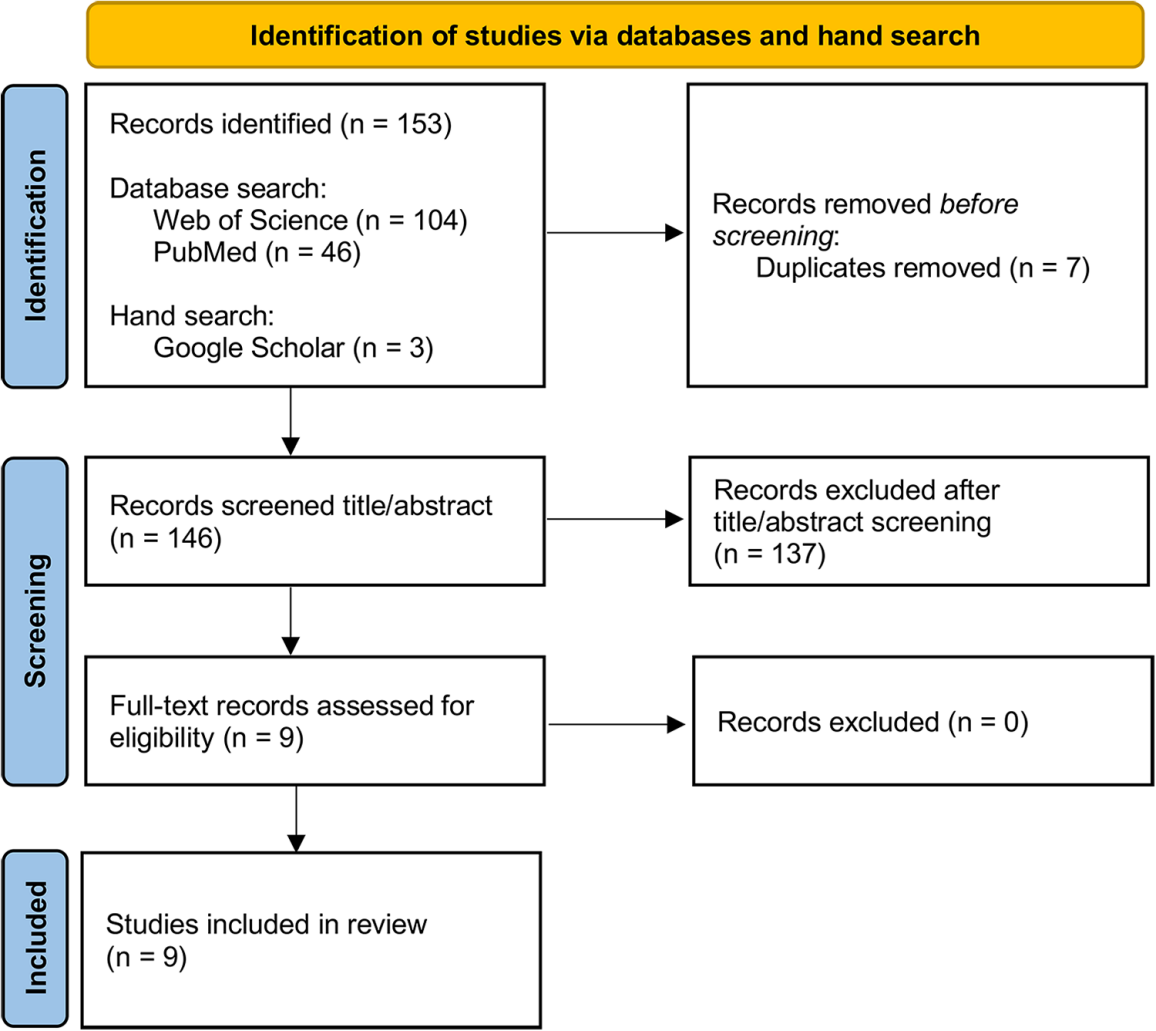
PRISMA flowchart representing the literature inclusion process.

Inclusion and exclusion criteria were defined regarding publication date, language, publication and article type, topic, region, and population (Table 1). Data regarding the studies' characteristics were extracted from full texts with data charting sheets (Supplementary Material 2). The PRISMA flowchart depicts the literature inclusion process (Figure 1). We identified 153 reviews describing factors influencing CR utilization. After exclusion of duplicates, 146 studies were screened for title and abstract. Nine studies were selected for full-text review and included for qualitative data synthesis.

**Table 1:** Inclusion and exclusion criteria.

Criteria	Inclusion	Exclusion
Publication date	2012–2023	
Language	• German • English	
Publication type	Research articles	Letters, Editorials, Abstracts
Article type	• Systematic reviews • Scoping reviews • Narrative reviews	
Topic-related	• Cardiology • Cardiac rehabilitation	• Other medical fields • Medication • Burden • Frailty • Risk factors
Country/region		• Reviews focusing exclusively on low-resource settings • Reviews focusing exclusively on African, Asian or North American contexts
Population	• Adults (+19 years) • Cardiac conditions and related co-morbidities	• Children • People with dementia, cancer or stroke

The included studies were published between 2012 and 2021. Six studies (30–35) were systematic reviews of quantitative research, two (8, 36) were systematic reviews of qualitative research, and one (37) was a scoping review. Eight studies (8, 30–33, 35–37) investigated the utilization aspects (referral, enrolment, adherence, completion), and one (34) the patients' engagement with physical activity. With regards to structuring the factors that influence CR utilization, two studies (32, 33) used a socio-ecological health model, and six (8, 30, 31, 34–36) used healthcare-related categories, such as the patient, provider and system level. One study (37) described the factors narratively without any given structure. Table 2 gives an overview of the study characteristics.

**Table 2:** Study characteristics.

First author	Pub. year	Review design	Included studies (n)	Focus on special patient population	Cardiac rehabilitation utilization aspects	Categorization of factors
Clark (36)	2012	Qualitative systematic review of qualitative or mixed-method studies	90	Not given	Attendance	Personal/Contextual
Clark (8)	2012	Qualitative systematic review of qualitative or mixed-method studies	34	Not given	Referral	Personal/Contextual
Clark (35)	2013	Systematic review of qualitative or mixed-method studies	62	Not given	Participation	Patient/Professional/System
Ruano-Ravina (30)	2016	Systematic review of cohort/cross-sectional studies	29	Not given	Participation, adherence	Gender/Age/Accessibility to CR/Employment status/Socioeconomic status/Comorbidities/Civil status
Supervia (31)	2017	Systematic review of interventional and cohort studies	24	Female patients	Referral, enrollment, completion	Patient/Provider/Societal/Environmental
Resurreccion (32)	2017	Systematic review of observational, interventional and qualitative studies	24	Female patients	Participation, dropout	Interpersonal/Intrapersonal/CR program/Logistical/Health system
Resurreccion (33)	2019	Systematic review of prospective cohort studies	43	Not given	Participation, dropout	Intrapersonal/Interpersonal/Clinical factors/Logistical/CR program/Health system
McHale (34)	2020	Systematic review of qualitative studies	12	Not given	Not given	Post-event communication and advice/Expectations of exercise-based CR
Vanzella (37)	2021	Scoping review of cohort or cross-sectional studies	20	Ethnic minority groups	Referral, enrollment, completion/adherence	Not given

### 3.2 Mapping factors influencing CR utilization to the BCW

The factors influencing CR utilization were mapped according to the BCW (10). The wheel can be considered a framework for understanding or targeting a specific behavior. It supports the design and implementation of evidence-based interventions by linking them to human behavior and therefore also lends itself for guiding retrospective analyses to these ends.

The BCW-hub includes the COM-B *Model of Behaviour*, where *Capability*, *Opportunity*, and *Motivation* influence each other and generate human behavior. The COM-B is encircled by the Theoretical Domains Framework (TDF). This framework aids in identifying influences on HCPs' behavior in implementing evidence-based care and studying the behavior of patient populations. The TDF in turn is based on 33 theories of behavior and behavior change, including the theory of planned behavior, social cognitive theory, and self-determination theory (38). These theories were deconstructed and simplified into 14 domains, such as *Knowledge*, *Beliefs about Capabilities*, and *Memory, Attention and Decision Processes* (39). The factors defined by the COM-B and the TDF can be tackled by nine *Intervention Functions*, i.e., activities aiming to influence a targeted behavior. These activities are, for example, *Education*, *Enablement*, *Persuasion,* and *Environmental Restructuring*. The *Intervention Functions* are encircled by seven policy categories, such as *Guidelines* and *Legislation*, not considered in this review.

The TDF was used to group factors according to specific behaviors that could hinder or lead to CR utilization. The final domains, according to the BCW, were summarized qualitatively.

## 4 Factors influencing CR utilization

**Figure 2:**
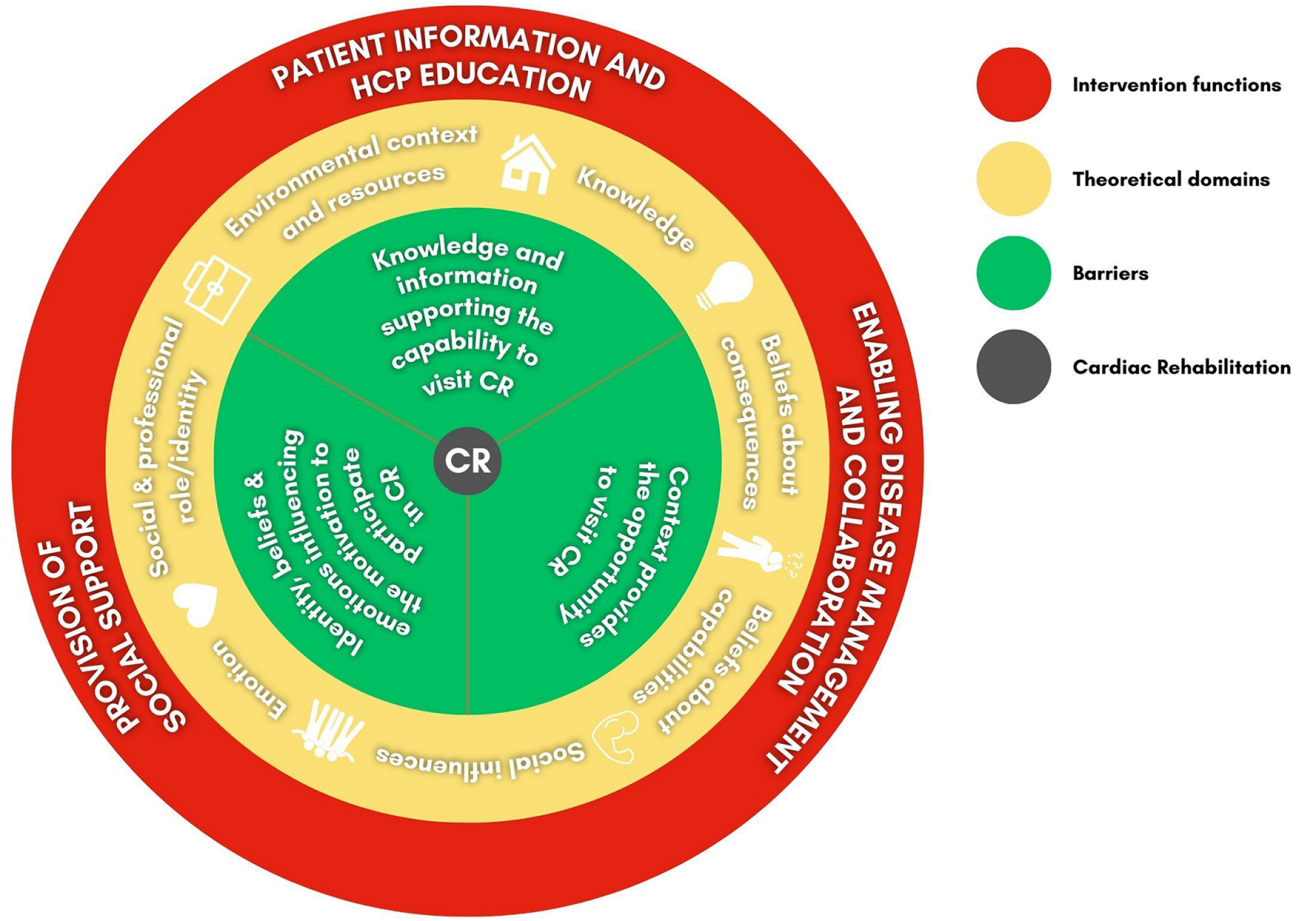
Factors influencing cardiac rehabilitation, theoretical domains and intervention functions according to the *behaviour change wheel* (BCW) domains (10) CR, cardiac rehabilitation; HCPs, healthcare professionals.

Figure 2 provides an overview of the factors that influence CR use. In the following, we present the factors influencing CR utilization according to the BCW domains (10).

### 4.1 Knowledge and information support patient capability to utilize CR

Patients' capability to take up CR was influenced by their knowledge about their disease and considering CR as fundamental for recovery, the received information, and the communication with HCPs.

Patients' knowledge about their disease and awareness regarding the role of CR in the recovery process facilitated CR utilization (8, 31, 34–37). The phase before the CR program started was characterized by an urgent information need. Therefore, timely information about CR can be helpful (8, 35). Receiving little information caused unawareness of CR and uncertainty about program benefits (31, 32, 34).

During CR, the educational components and the opportunity to ask questions facilitated adherence, whereas lack of interaction with the HCPs was a barrier (32, 37). Receiving individual exercise advice, monitoring the recovery progress, and assessing symptoms were linked to feelings of security and safety. The supervision supported patients who perceived the risk of CVD as unpredictable, inevitable, and uncontrollable (37). In contrast, language differences resulting in communication difficulties with HCPs and a lack of understanding of written and verbal information were barriers to CR utilization (37).

### 4.2 Patient identities, beliefs and emotions influence their motivation to participate in CR

Personal factors, patients' self-perception, and comparisons with fellow participants influenced patients' motivation to participate in CR. Emotional barriers and a sense of control over their condition also significantly determined their willingness to take up CR.

Gender, age, and occupation were described as influential factors in patients' motivation to attend CR. For example, patients who felt too old to exercise were less likely to participate in CR. Especially in female patients, placing family obligations or occupational demands above health needs was a barrier to CR uptake (30, 33, 35, 36).

Two studies described a fitness identity (34, 36) as a relevant factor for taking up CR: Patients who had the self-perception of already being active enough or who underestimated the severity of their illness were less likely to participate (32, 34–36). Additionally, comparing oneself with other CR participants influenced adherence. Patients who perceived themselves as more fit than their CR fellows were more likely to quit CR (34).

Moreover, feelings and emotions were strongly related to CR utilization. Feeling too sick, too old, overwhelmed, and out of control were mentioned as barriers (33–35, 37). Uncertainty and anxiety about exercising and being unable to address these feelings in the native language also led to non-participation (35). Another barrier was the belief that CR would not make any difference to the current health status; thus, attendance was not considered necessary (32, 33). Negative experiences reinforced this perception (31, 32, 34–36), such as missing social support during exercises or HCPs being too judgmental (34, 35). Irrational health beliefs, for example, the belief in being capable of managing the CVD by oneself, were seen as a barrier to CR. In contrast, a high sense of control over the heart condition was a facilitator (34, 36). Moreover, it was a facilitator for the uptake of CR when patients were aware of the health benefits and recognized CR as crucial for their recovery (34). Motivation was also reinforced by information about the aims and objectives of the CR program. The prospect of a supervised environment and HCPs supporting the setting of appropriate exertion levels and rehabilitation goals positively influenced CR uptake (34). Generally, HCPs' encouragement was essential for CR adherence (35).

### 4.3 Healthcare system factors and personal resources influence patients' opportunities to utilize CR

Patients' opportunities to attend CR were influenced by healthcare system factors such as ineffective CR referral processes and HCP awareness, as well as personal resources including logistical challenges and comorbidities; additionally, patients' relatives played a dual role as facilitators and inhibitors in CR utilization.

The patient's social context and healthcare system barriers were repeatedly described as influential factors in CR utilization (8, 30–37). First, the lack of CR referrals in hospitals and a constricted information flow across healthcare sectors hindered initiating the referral process (8, 30, 33, 37). Also, when HCPs were unaware of the indications and did not know that the patient was suitable for CR, this was a barrier to referral (8). Within the CR settings, programs that were unresponsive to the needs of ethnic minorities or women hindered the ongoing uptake of CR (34, 35). For example, when exercising with men was considered sinful for religious reasons, CR programs with mixed-gender classes were considered inappropriate (34).

Regarding personal resources, logistic barriers hindered the uptake of CR, such as a lack of transport possibilities, being a non-driver, and living in a rural setting with poor public transport links (30, 32, 35–37). Moreover, physical barriers were mentioned; for example, a high disease severity or recovery from surgery prevented patients from attending CR and focusing on physical activity. Besides, comorbidities, such as depression, musculoskeletal diseases, obesity, and diabetes, were related to non-attendance. Psychological factors, such as symptom-related pain or anxiety, were also described as barriers (30, 31, 33, 34, 37). Patients with fatalistic health beliefs due to religious reasons, for example, being fated to have heart disease, were less likely to participate in CR (32, 37).

The patients' families strongly influenced CR utilization (31–33, 35–37). On the one hand, relatives were described as facilitators when supporting patients in risk factor management during CR (36). On the other hand, families could also represent a barrier to CR attendance by withholding information to prevent patients from becoming distressed about their CVD (37).

## 5 Discussion and considerations for future innovations aiming to support CR utilization

Based on the results of the BCW analysis regarding factors influencing CR utilization, we identified seven relevant theoretical domains (10) (Figure 2). These domains are intertwined and influence patients' behavior in the context of CR utilization:

• Environmental context and resources
• Knowledge
• Beliefs about consequences
• Beliefs about capabilities
• Social influences
• Emotion
• Social and professional role/identity

In the following, we propose three intervention functions (10) that are especially important when it comes to addressing the behavioral factors stated above: *patient information and HCP education*, *enabling disease management and collaboration*, and *provision of social support for cardiac patients* (Figure 2). We discuss how future innovations could incorporate these functions and SDM to increase CR utilization. Table 3 provides an overview of evidence-based considerations for DHTs designed to support CR uptake.

**Table 3:** Considerations for future digital health technologies aiming to support cardiac rehabilitation utilization.

Aim	Recommendations
Providing information for patients and HCPs	• Combine educative elements with monitoring features, enabling video conferences or counseling with (virtual) HCPs • Provide evidence-based, clear, personalized information about CR health benefits • Consider individual factors (gender, age, knowledge level, diagnosis) for personalized content • Allow HCPs and patients to jointly select preferred information in alignment with medical guidelines and patient needs • Implement decision aids to empower patients in SDM • Tailor educational content to provide timely and targeted support at critical moments during rehabilitation
Enabling disease management and collaboration	• Provide virtual platforms for CR programs to overcome accessibility barriers by offering real-time monitoring, exercise guidance, and interaction with HCPs • Incorporate gamification features, virtual reality games and persuasive elements to virtual CR programs • Implement monitoring features and wearable devices to facilitate self-management and to increase self-efficacy • Provide real-time data for patients and HCPs, enabling timely support and intervention • Tailor support from diagnosis through CR to long-term post-rehabilitation care • Make SDM integral, allowing patients to shape their rehabilitation journey, for example through joint goal setting with their HCPs
Enhancing social support	• Explore mobile applications that track contextual, experiential, and behavioral data to initiate co-responsibility between patients and their relatives • Establish and foster online platforms for cardiac patients to exchange personal stories and receive social support from relatives • Leverage the power of peer experiences to inform patients about the benefits of CR, for example, trough peer testimonials

CR, cardiac rehabilitation; HCPs, healthcare professionals; SDM, shared decision-making.

### 5.1 Providing patient information and HCP education

Evidence indicated that a central intervention function should address patients' lack of knowledge about their disease and the role CR can play in their recovery. Receiving little information caused unawareness about program benefits, representing a barrier to participating in CR (8, 31, 34–37). We conclude that information about the benefits of CR and preparation regarding what to expect during the program could help reduce patients' uncertainty about their recovery. With information emphasizing the necessity of CR to reduce the likelihood of further cardiac events, patients are prevented from developing inaccurate assumptions and beliefs regarding their benefits from CR. Further research indicates that especially unemployed women would benefit from tailored educational interventions (40).

Digital health holds great promise to provide patients with evidence-based, easily accessible educational content. Such content will help patients understand the importance of CR and the associated lifestyle changes. Related work describes DHTs incorporating educative elements and providing patient information to increase patients' knowledge regarding their condition. For example, disease-specific symptoms in electronic bookshelves, e-learning programs, or digital transcripts of the patient-HCP encounter have been implemented (41–43). Other DHTs combine educative elements with monitoring features, providing the possibility for video conferences or counseling with (virtual) HCPs (42, 44).

Kim et al. (45) developed a support tool that provides patients facing bone marrow transplants with personalized, clinically validated information about possible outcomes of treatment options. They investigated patients' preferences regarding the presentation of outcome likelihoods with survival calculators and found that sense-making regarding the health condition and emotional support was crucial for patients. In particular, they expressed a need for structured, personalized information (45). Related work shows that the need for evidence and personalized, credible information is also present in cardiac patients (23, 46). Sankaran et al. (41) demonstrated how a single DHT can address these needs. They prototyped a system through which HCPs and patients can jointly select preferred information conforming to medical guidelines, patient needs, and pathways. The information was adapted to the patient's level of knowledge for a remote CR program and chosen in an SDM process between the HCP and the patient (41).

We also found that HCPs' knowledge gaps and unawareness can be a barrier for CR referral (8). Clinical decision support has the potential to address this barrier, for example, by raising awareness about regional services, highlighting appropriate indications, and facilitating the CR referral process. Abidi et al. (47) investigated how such a system can support family physicians in evidence-based treatment decisions. Based on current medical evidence, the system provides advice on monitoring risks and contraindications for multiple cardiac conditions. During the clinical encounter, it also provides the opportunity to note patients' preferences to facilitate SDM (47).

Decision aids within technologies potentially empower patients to engage in SDM with healthcare providers. Patients are well-informed about their options and able to actively participate in selecting treatment plans that align with their goals and values. The premise of practical use is that DHTs provide personal content tailored to individual factors, such as gender, age, level of knowledge, and diagnosis (23, 46). Interventions should also respect patients' preferences regarding their degree of involvement in SDM and tailor the support accordingly. DHTs can provide timely and targeted support to address individual patients' challenges at critical moments in their cardiac rehabilitation journey.

### 5.2 Enabling disease management and collaboration along the cardiac patient pathway

Limited personal resources, for example, lack of transportation possibilities, limited physical fitness due to comorbidities, and pain or anxiety, hindered patients from participating in CR (30, 32, 35–37). Therefore, a second relevant intervention function works to increase the patients' self-efficiency in managing their condition and enable collaboration with their HCPs.

Remote CR programs have gained popularity within the last few years. They represent an option to overcome accessibility barriers (48). Given the growing acceptance of telehealth as an alternative to center-based rehabilitation, CR programs may become more accessible through virtual platforms (49). Digital CR programs could offer real-time monitoring, exercise guidance, and interaction with HCPs from the patients' homes. Previous research has already highlighted how remote CR could positively affect cardiac patients' cardiorespiratory fitness (50). Future DHTs can contribute to this with gamification features and persuasive elements, impacting patients' adherence and motivation. For example, Geurts et al. (51) developed an immersive virtual reality game to motivate patients to exercise by guided cycling in a safe and enjoyable environment. Gatsios et al. (52) also suggested a combination of gamification and virtual coaching to improve adherence to home rehabilitation programs. In order to support behavior change and a sustainable healthy lifestyle, Wong et al. (53) propose a serious game with fictive scenarios to encourage patients to reflect on their values and make conscious health-related decisions. For example, patients could earn rewards or incentives for meeting specific rehabilitation milestones, making digital CR programs more engaging and enjoyable.

Furthermore, DHTs incorporating self-monitoring features can facilitate self-management and collaboration between patients, HCPs, and relatives. Salamah et al. (54) provide an example of a mobile application allowing patients with autoimmune diseases to track symptom progression, vital information, and laboratory results. Further, the integration of wearable devices could enable continuous monitoring of a patient's cardiac condition at a much more fine-grained and adequate level. These devices could provide patients and healthcare providers with real-time data, ensuring they receive timely support and intervention when needed. Innovations in outcome tracking will enable patients to monitor their progress over the long term, promoting accountability and motivation. Patients may also receive personalized recommendations based on their tracked data.

Future DHTs should prioritize the development of highly personalized care pathways that cater to individual patient needs and preferences. Pathways should also offer tailored support from the point of diagnosis through CR and long-term post-rehabilitation care. SDM will be integral, enabling patients to actively shape their recovery journey, from choosing the preferred rehabilitation program to adjusting it based on their evolving needs and preferences. Therefore, DHTs adapt their content to not only guiding patients through the decision-making process about CR participation but also providing support for emotional well-being and facilitating a sustainable lifestyle change.

Related work already demonstrated how personalized decision support could be supported by digital systems. Peleg et al. (55) introduced a personalized evidence-based decision-support system for HCPs and patients with chronic diseases. The system incorporates a module to elicit patients' preferences and psycho-social context. It provides real-time personalized recommendations combined with medical guidelines and informs the SDM process during a patient-HCP encounter (55). Regarding personalized goal-setting, Chaudhry et al. (56) developed a DHT for community-dwelling older adults with chronic multimorbidity. It supports care workers and residents in setting health goals jointly.

### 5.3 Enhancing social support from relatives and peers

Barriers to CR utilization showed that patients' families played a significant role as they can support or hinder patients' uptake of CR (31–33, 35–37). Peer comparisons and the perception of not fitting into the group of people who need CR were also barriers (34, 36). Cardiac patients' need for social support is in line with existing evidence (57, 58) and, hence, should be incorporated by DHTs as a third intervention function.

Related work demonstrates how social support could be enhanced digitally. Jansen et al. (59) investigated how co-responsibility between bariatric patients and relatives could be initiated by a mobile phone application comprising features to track contextual, experience and behavioral data. They found that shared routines of relationships could facilitate lifestyle change. However, patients' partners were not aware of their role and lacked knowledge about how to support (59). Coull et al. (60) researched cardiac patients' attitudes towards physical activity and found that social support from family and friends was crucial for maintaining an active behavior. Patients valued an online platform for exchanging personal stories with peers. The feeling of helping peers by sharing experiences and knowledge was also appreciated.

Future DHTs could enhance development of virtual peer networks specifically for CR patients. These networks could offer support, motivation, and sharing of personal experiences to encourage adherence to the program. Research has demonstrated the positive impact of peer support on patients' ability to retain information, boost self-efficacy, and enhance overall well-being (58, 61). Peers can provide emotional support and help individuals navigate periods of uncertainty, for example, through testimonials sharing experiences (62). It is conceivable that upcoming innovations will incorporate such social aspects into SDM, e.g., leveraging the power of peer testimonials to inform patients about the benefits and experiences of cardiac rehabilitation. Digital health platforms could foster engagement within a community of CR patients, facilitating discussions, support, and knowledge sharing. This sense of belonging to a community can be a powerful motivator.

## 6 Conclusion

This review represents the first step towards a more patient-centered and need-based development for DHTs to increase CR utilization. Our synthesis of evidence provides barriers and facilitators to CR and possible digital interventions according to the BCW.

The patient's capability to attend CR is influenced by disease knowledge, awareness of the benefits of CR, and interactions with HCPs. Additionally, contextual factors such as referral processes, HCPs' awareness, and patients' resources, including logistical challenges, influence their opportunity to participate in CR. The motivation to engage in CR is affected by patients' emotions, self-perception of fitness and control over the cardiac condition, and peer comparisons. Based on this, we found that patient information, HCP education, enablement of disease management, collaboration along the patient pathway, and enhancing social support from relatives and peers are relevant intervention functions. To conclude, we considered how future DHTs could incorporate these functions.
