# Supplementary material for: Targeting behavioral factors with digital health and shared decision-making to promote cardiac rehabilitation—a narrative review
Source: Front Digit Health. 2024 Feb 23;6:1324544. doi: 10.3389/fdgth.2024.1324544 (PMC10920294; doi:10.3389/fdgth.2024.1324544)
Supplement: Supplementary file 1 [file Datasheet1.pdf]

Supplementary material 1: Search protocol

Database	Date	Search string	Records
PubMed	25.09.23	((((((((((((barriers) OR (facilitators)) OR (determinants)) OR (factors)) AND ((((((((((utilization) OR (utilisation)) OR (usage)) OR (access)) OR (referral)) OR (enrolment)) OR (enrollment)) OR (participation)) OR (adherence)) OR (completion))) AND (cardiac rehab*)) NOT ((((LMIC) OR (Africa)) OR (Asia)) OR (North America)))) NOT ((pediatric) OR (paediatric))) NOT (frailty)) NOT (dementia)) NOT (cancer)) NOT (medic*)) NOT (burden)<br><br>Filters: Review, Systematic Review, Adult: 19+ years, from 2012 - 2023	46
		Records after Title/Abstract-Screening	4
		Records after Full-Text Screening	4

Database	Date	Search string	Records
Web of Science	27.07.22	((((((((((((barriers) OR (facilitators)) OR (determinants)) OR (factors)) AND ((((((((((utilization) OR (utilisation)) OR (usage)) OR (access)) OR (referral)) OR (enrolment)) OR (enrollment)) OR (participation)) OR (adherence)) OR (completion))) AND (cardiac rehab*)) NOT ((((LMIC) OR (Africa)) OR (Asia)) OR (North America)))) NOT ((pediatric) OR (paediatric))) NOT (frailty)) NOT (dementia)) NOT (cancer)) NOT (medic*)) NOT (burden)<br><br>Filters: Reviews, from 2012 - 2023	104
		Records after Title/Abstract-Screening	6
		Records after Full-Text Screening	6

Records after initial search	10

Records after removing duplicates	6

Hand search	Date	Search terms	Records
Google Scholar	26.07.22	Barriers, cardiac rehabilitation	3

**Total included studies**	**9**
