# Supplementary material for: Targeting behavioral factors with digital health and shared decision-making to promote cardiac rehabilitation—a narrative review
Source: Front Digit Health. 2024 Feb 23;6:1324544. doi: 10.3389/fdgth.2024.1324544 (PMC10920294; doi:10.3389/fdgth.2024.1324544)
Supplement: Supplementary file 2 [file Datasheet2.pdf]

Barriers to cardiac rehabilitation utilization

A review of reviews

Data charting sheets

Influences on attendance at cardiac rehabilitation programs after referral (1)		
First author	**Clark,** AM	
Publication Year	2012	
Country	Canada	
Study Design	Qualitative Systematic Review	
Aims/objectives	To examine factors and processes that influence patient decisions to attend CR programs and similar secondary prevention systems	
Data bases searched	10	
Number of included studies	90	
Inclusion criteria	• Studies conducting primary qualitative research or with mixed-methods-design<br>• Adults >18 yrs.<br>• Publication year >1995	
Data synthesis	Meta-ethnographic approach	
Key findings	Personal barriers	• **Low insight + knowledge:** regarding the nature of programs; patients perceived that CR would not be beneficial generally or people like them, receiving little information from HCP on program content + benefits; patients feel little encouragement to attend; HCPs find patients having little interest in programs<br>• **Beliefs about heart disease:** attempts at risk reduction are perceived by patients to be futile; CHD seen as unpredictable, inevitable + uncontrollable (low sense of control over their future health)<br>• **Negative views of services/health system**: HCPs are seen to disrespect patients, provide insufficient time for consultation, give narrow/mixed messages about recovery; poor/insensitive communication; long wait times after referral; HCPs being unresponsive to the needs of particular populations such as older adults or ethnic minorities<br>• **Self + identity**: patients seeing themselves as different from the type of people who should participate (for "old" people); services being not needed, unlikely to benefit, conflicting with priorities; patients engaged in avoidance strategies to downplay the need to attend; ADLs are seen as health in terms of physical activity; anxiety; pain; recovery from surgery; other illnesses<br>• **Financial + work constraints**: low income; uncertain employment; competing occupational demands; no flexible home-based program offered<br>• **Demands on women**: struggled to meet social + financial costs of participation; domestic/family demands; receiving little support from wider community
	Contextual barriers	• **Long distances to services:** long travel distances from rural settings; poor transport links<br>• **Lack of support from family:** overprotecting families; families take charge of risk factor reduction
Discussion	• Wide range of factors influencing CR attendance was found	

	• Medical reasons for low attendance such as symptoms or comorbidities were invoked far less often as reasons for nonattendance<br>• High influence of psychosocial factors on decisions to attend CR
Limitations	• 1/3 of the studies were conducted in the UK<br>• Constrained by quality (moderate) and scope of existing literature<br>• Only high-income countries included
Recommendations	• Professionals from multidisciplinary teams should adapt their support to the individual (by using principles of adult learning)<br>• Interventions including “decision-aids” to ensure that all issues have been raised<br>• Providing accurate and personalised feedback<br>• Fostering patients’ sense of individual control over their CHD<br>• Engage patients AND their families

**Factors influencing referral to CR and secondary prevention programs** (2)			
First author	**Clark,** AM		
Publication Year	2012		
Country	Canada		
Study Design	Systematic Review		
Aims/objectives	To explore factors and complex processes that influence referral as opposed to pre-identified predictors of referral		
Data bases searched	11		
Number of included studies	34		
Inclusion criteria	Qualitative research/mixed-methods design		
Data synthesis	Meta-ethnographic approach		
Key findings	Patient barriers	Professional barriers	Systems barriers
	• **Lack of consistent information + encouragement:** patients reported that they received limited or no information/encouragement from physicians + HCP regarding possible participation in programs; Patients having to initiate their own referral by asking HCP or by calling programs on their own behalf<br>• **Patient memory:** patients could not remember having been given information about programs while in hospital; especially when information was given prior to surgery or when patients found care chaotic + confusing → uncertainty about CR or confusing CR with exercise test/outpatient appointment	• **Physician as gatekeeper:** physicians as only type of HCP referring patients; patients waiting for a formal invitation to participate; patients have to initiate their own referral to a program through a physician;<br>• **Lacking knowledge** about content + benefits of programs; about indications + contraindications of exercise<br>• **Patients felt not being included into decision-making process:** women felt as “silent players”<br>• **Patients being denied** a referral by a physician or told that their condition was not bad enough → patients being dependent on physicians to negotiate referrals<br>• **Patients were viewed by HCPs** as having low interest or motivation for participating	• **Local territoriality**: providers reported not referring patients to programs out of fear that these services would replace physician managed care<br>• **Programs with different modes of delivery**: could be viewed as being in competition for patients<br>• **Poor communication across healthcare settings** with regard to sharing patient information (laboratory + exercise tolerance tests)<br>• **Absence of physician reimbursement** for referral + lack of time to refer within competing workload priorities<br>• **Programs lacking resources** + thus capacity to meet patient needs<br>• **Physicians having less capacity** to refer due to time constraints<br>• **Timing of referral + enrolment:** difficulty of providing patients with a

			referral prior to hospital discharge; hospital stays considered too short for patients to receive a referral from HCP
Discussion	• Most patients are not referred for avoidable reasons<br>• Systems-based solutions should promote shared care<br>• Timely and automated referral should be implemented<br>• Educational outreach<br>• In the studies reviewed, referral was almost exclusively discussed in relation to hospital-based programs with little recognition of alternative types<br>• Despite increased availability of various referral mechanisms, some patients may not attend CR due to complex social + personal factors<br>• Eligible patients should be referred to CR for clinical and ethical reasons irrespective of whether patients indicate they will subsequently participate		
Limitations	• Constrained by the quality of the included studies (small samples, urban medical centres)<br>• Few studies addressed non-hospital programs		

**Participation and adherence to CR programs** (3)	
First author	**Ruano-Ravina,** A
Publication Year	2016
Country	Spain
Study Design	Systematic review
Aims/objectives	To determine, which factors influence participation and adherence rates in CR programs in patients with AMI
Data bases searched	3
Number of included studies	29
Inclusion criteria	• Sample size >100<br>• Study design: systematic reviews and meta-analyses, case-control studies and cross-sectional studies<br>• Patients: AMI, coronary heart diseases<br>• Publication date >2004
Data synthesis	n.a.
Key findings	Barriers related to participation and adherence:<br>• **Gender**: women participate less in CR than men do<br>• **Age**: older individuals participate less than younger ones<br>• **Employment status:** dropout because of return to work<br>• **Income:** higher income was associated with higher participation and higher education; higher socioeconomic status may lead to higher adherence<br>• **Comorbidities:** suffering from depression, feeling too ill to participate<br>• **Civil status:** living alone or being single<br>• **Other aspects:** being a smoker, lack of interest or the perception that the program was not going to be useful<br>• **Accessibility**: individuals living farther from the nearest CR centre; not owning means of transportation, or not having a driving license; difficulties in transportation could lead to less adherence
Principal findings and discussion	• Factors appear homogeneously in most studies<br>• Studies conducted in USA, UK, Canada, Belgium, Denmark, Germany, Australia<br>• Participation and adherence patterns may differ between EU and USA due to different health coverage systems and costs<br>• Factors affecting participation are usually the same in different locations<br>• Subgroup-specific approaches seem to be needed in order to increase CR participation in women, elderly, and individuals without transportation possibilities
Implications for future research	• Qualitative studies can provide interesting inputs in specific settings and should be used to study patients' and cardiologists' attitudes towards CR programs<br>• Specific CR approaches needed for subgroups
Limitations	• No meta-analysis due to the heterogeneity of the studies included

AMI = acute myocardial infarction

**Factors associated with non-participation in and dropout from CR** (4)		
First author	Resurrección, DM	
Publication Year	2019	
Country	Spain	
Study Design	Systematic review of prospective cohort studies	
Aims/objectives	To review factors specifically associated with non-participation (NP) in and/or dropout from CR	
Number of data bases searched	6	
Number of included studies	43	
Inclusion criteria	Prospective cohort studies	
Data synthesis	Data extraction sheets	
Key findings	Intrapersonal factors	• Older age or young age associated with NP<br>• Female sex (four times higher odds of NP)<br>• Male sex higher dropout rates<br>• Vulnerable socioeconomic situation<br>• Comorbid conditions: were associated with up to nearly twice the odds of NP + with higher dropout rates<br>• Depression: positive relationship between depression + anxiety with dropout<br>• Low self-efficacy for managing disease was associated with NP<br>• Not feeling the need for CR
	Clinical factors	• Smoker: nearly 2x the odds of NP + 3x odds for CR dropout<br>• Higher BMI: increased risk for NP + dropout<br>• Comorbidities: Diabetes, obesity, not having controlled cholesterol were associated with NP
	Interpersonal factors	• Being single<br>• Being unemployed or retired: higher rates of NP<br>• Being employed: higher odds of NP and dropout<br>• Low practical + social support have been identified as factors associated with NP
	Logistical factors	• Longer travel times, being a non-driver, lack of transport, living in a rural area or in a geographically inaccessible area were associated with NP
	Cardiac rehab	• Attending 2x/week was associated with higher odds of dropout than attending 3x
	Health system factors	• Lack of a referral or having a low strength of endorsement from physicians was associated with NO<br>• Long intervals between doctors' visit: 4x greater odds of dropout
Discussion	• Clinical factors, logistical factors and health system factors were the main factors assessed for NP in + dropout from CR<br>• Studies from USA, Canada, Europe, Oceania	

	• Several factors with scarce evidence: ethnicity, employment, practical support and illness beliefs<br>• Age, gender and employment: controversial for NP + dropout<br>• Findings argue in favour of automatic referral regardless of cardiovascular diagnosis and sociodemographic factors<br>• Combination of inpatient CR + eHealth or community resources would decrease NP + dropout rates due to better adoption to patient profiles
Implications for future research	• Systematic referral as an option to address non-participation
Limitations	• Heterogeneity<br>• Half of studies provided non-adjusted results<br>• Hospital-based settings only

NP = *non-participation*

<table>
<tr><th colspan="3">Factors influencing participation in CR after referral and initial attendance (5)</th></tr>
<tr><td>First author</td><td colspan="2">Cark, AM</td></tr>
<tr><td>Publication Year</td><td colspan="2">2013</td></tr>
<tr><td>Country</td><td colspan="2">Canada</td></tr>
<tr><td>Study Design</td><td colspan="2">Qualitative systematic review and meta-synthesis</td></tr>
<tr><td>Aims/objectives</td><td colspan="2">To examine the process and factors that influence patient decisions to complete programmes after referral and initial programme access to build a basis for future interventions aiming at promoting higher participation rates in CR</td></tr>
<tr><td>Number of data bases searched</td><td colspan="2">11</td></tr>
<tr><td>Number of included studies</td><td colspan="2">62</td></tr>
<tr><td>Inclusion criteria</td><td colspan="2">• Studies reporting primarily qualitative data (wholly or as part of mixed-methods designs)
• Adults >18 yrs.
• Secondary prevention or CR after referral
• Publication date >1995
• Definition “participation”: findings related to any process, phenomena, or construct that pertains to participation in a secondary prevention or CR programme at referral, uptake or at participation stages”</td></tr>
<tr><td>Data synthesis</td><td colspan="2">Qualitative meta-synthesis (iterative approach)</td></tr>
<tr><td rowspan="2">Key findings</td><td>Individual factors</td><td>Theme Identity and the self
• Patients struggling to assimilate their diagnosis
• Programmes were seen to people who were older, sicker, less fit, take more risks, and need goals prescribed for them → program not beneficial for themselves
• Women: concern that participation would increase stress on other family members
Theme Negative views and reactions to health care
• Negative experiences of programme or programme providers
• Received information in CR was inconsistent, badly timed, cajoling
• Health professionals were too judgmental and lacking in cultural sensitivity
• Services poorly organized, to narrowly focused without including the need of the individuals, not beneficial and less effective than surgery
• Women: missing social support and encouragement by services/staff, preferring individual care over group-based sessions
Theme Views and reactions to heart disease
• Anxiety: feeling overwhelmed, out of control, public exercising, social interaction in the rehab centre
• Denial of diagnosis
• Women: process of adjustment after the diagnosis of heart disease, uncertainty, desire to return to normality</td></tr>
<tr><td>Contextual factors</td><td>Theme Distance and transport issues</td></tr>
</table>

		• To rely on public transport or on family members<br>• Women: living in a rural setting, distance, and time<br>Theme *Lack of family support*<br>• Relying on family members to participate in programs<br>• Women who were caregiver for their partners or provided income to other family members<br>Theme *Gender roles*<br>• Programmes seen as "men's clubs" that insufficiently recognize the contemporary social and occupational roles of women<br>• Conflict with women's occupational demands<br>• "Domestic responsibilities" as childcare, housework, family life: Perceived responsibility to other family members were often placed by women before their health needs<br>• Services failing to be sufficiently inclusive of patients' different languages, cultures, and clothing preferences while exercising
Principal findings and discussion	• Participation was more fundamentally influenced by social dimensions of services related to user experiences, conceptions (of identity, the services, and the disease) and contextual factors<br>• Social experience of attending programmes was highly valued by patients and seen to be beneficial<br>• Most patients see CR as being intended for patients who are less healthy than they are<br>• Perceptions and knowledge of programme benefits and risks of non-participation appear to influence participation less	
Implications for future research	• Gender-sensitivity: Interventions should be adapted to women's needs and must seek to supplement the information content of CR with approaches that harness the social elements of CR<br>• Interventions should encourage patients to see their own values represented in CR programmes and the professionals providing them<br>• Social marketing approaches: Interventions should use social marketing to link CR content to a wide range of types of patients in terms of age, sex, and fitness levels<br>• Mobilize family support: Support and education of relatives regarding their role in facilitating the health benefits that can accrue from CR	
Limitations	• Publication date 1995 or later<br>• Limited age or sex-based analysis and lack of theory/concepts to interpret or understand the influence of gender/ethnicity in detail<br>• Older adults were underrepresented in the studies	

**Barriers to CR in ethnic minority groups** (6)		
First author	Vanzella, L	
Publication Year	2021	
Country	Brazil	
Study Design	Scoping review	
Aims/objectives	To identify barriers to CR reported by people from ethnic minorities	
Number of data bases searched	6	
Number of included studies	20	
Inclusion criteria	• Peer-reviewed literature<br>• Other languages than English or Portuguese<br>• Studies including patients from ethnic minorities with CVD eligible to participate CR<br>• Definition “ethnic minority”: a group of people of a particular nationality living in a country or area where most people are from a different nationality	
Data synthesis	Thematic analysis	
Key findings	Barriers to CR referral	• Lack of CR knowledge<br>• Unawareness of CR programs<br>• Information sharing problems (patient-provider communication): Lack of discussion about CR<br>• Language proficiency: Documents with information not discussed with providers or not provided in native language
	Barriers to enrolment	• Lack of family support: withhold of information from patients to prevent them from becoming alarmed or distressed about their CVD<br>• Language: Lack of understanding of verbal and written instructions<br>• Information material not adapted to the language, cultural, and religious needs<br>• Lack of interaction to the CR team and communication about feelings and disease-related information<br>• CR knowledge: patients being unaware of the role of CR as no discussions were initiated by providers<br>• Logistical barriers: lack of transport, lack of time, distance to the CR program, work conflicts, long waiting times to start CR<br>• Fatalistic health beliefs (health problems chosen by God)<br>• Individual perceptions: being too old to change habits or exercise, limited understanding of their CVD, cardiac misconceptions<br>• Financial difficulties
	Barriers to adherence	• Logistical barriers<br>• Lack of family support

		• Individual perceptions: lack of motivation, feeling CR is not helpful, not feeling comfortable with the exercises, feeling vulnerable and hopeless about the future<br>• Language and communication difficulties<br>• Lack of knowledge about CR and cardiac condition<br>• Religion: being fated to have CVD, dietary habits, lack of cultural adaption of messages/info<br>• Psychological status, socioeconomic status
Principal findings and discussion	• Inadequate health literacy, lack of cultural adaption, limited provision of interpretation services, lack of translated and customized educational materials, short consultation times, resource constraints<br>• Especially: language, logistical aspects, lack of CR knowledge, individual perceptions, culture, lack of family support<br>• Referral as dependent upon patients' characteristics, providers' practice, and health system characteristics	
Implications for future research	• Overcome language barriers: appropriate translation of info material<br>• Dissemination of patient education materials or motivational letters in patients' first language<br>• Training for providers to improve cultural awareness and communication skills<br>• Encouragement to provide in-hospital discussions about CR<br>• Extra support for ethnic minorities (cultural, psychological, practical, financial)<br>• Liaison strategies: Automated referral in combination with an in-hospital discussion with providers	
Limitations	• Generalizability<br>• No RCT included	

<table>
<tr><th colspan="3">CR for women (7)</th></tr>
<tr><td>First author</td><td colspan="2">Supervia, M</td></tr>
<tr><td>Publication Year</td><td colspan="2">2017</td></tr>
<tr><td>Country</td><td colspan="2">USA</td></tr>
<tr><td>Study Design</td><td colspan="2">Systematic Review</td></tr>
<tr><td>Aims/objectives</td><td colspan="2">• To identify gender-related barriers to CR participation
• To rate solutions to the gender-related gap in CR participation</td></tr>
<tr><td>Number of data bases searched</td><td colspan="2">Min. 5</td></tr>
<tr><td>Number of included studies</td><td colspan="2">54 (24 studies regarding barriers and 31 studies regarding potential solutions)</td></tr>
<tr><td>Inclusion criteria</td><td colspan="2">• RCT, controlled clinical trials, observational studies
• Adults >18 yrs.
• Quantitative research</td></tr>
<tr><td>Data synthesis</td><td colspan="2">• Systematic review software (Covidence)
• Scoring solutions according to the AHA for level of evidence and strength of recommendation</td></tr>
<tr><td rowspan="3">Key findings</td><td>Patient-level factors</td><td>• Co-morbidities (depression, diabetes, obesity…)
• Lack of information on or familiarity with CR
• Negative beliefs and perceptions about CR
• Lack of CR awareness
• Perception of exercise as being tiring or painful</td></tr>
<tr><td>Provider-level factors</td><td>• Supportive endorsement is helpful for CR participation
• Lack of written referral necessary for participation</td></tr>
<tr><td>Societal/Environmental factors</td><td>• Transport problems
• Family obligations and responsibilities
• Lack of insurance and financial concerns
• Lack of social support from family and friends
• Individuals from underrepresented minority groups
• Low education level</td></tr>
<tr><td>Principal findings and discussion</td><td colspan="2">• Barriers identified reflect a complex array of demographic, socioeconomic, medical, and societal challenges
• Non-modifiable factors vs. modifiable
• Increased public awareness on cardiac diseases through campaigns should be supported</td></tr>
<tr><td>Implications for future research</td><td colspan="2">• Alternative delivery models for women must be taken into account
• Uptake of gender-specific research</td></tr>
<tr><td>Limitations</td><td colspan="2">• Quantitative research studies only
• Low availability of gender-based solutions to CR referrals</td></tr>
<tr><td colspan="3">Previous experience of physical activity influences engagement with CR (8)</td></tr>
</table>

First author	McHale, S	
Publication Year	2020	
Country	UK	
Study Design	Systematic review	
Aims/objectives	• To examine qualitative evidence systematically<br>• To describe patients' perceptions and experiences	
Number of data bases searched	4	
Number of included studies	12	
Inclusion criteria	• Publication date 1990-2017<br>• Adults >18 yrs. eligible to attend CR with ACS	
Data synthesis	• Thematic synthesis methodology (Thomas and Harden)	
Key findings	Barriers related to post-event communication + advice	• Communication regarding CR primarily focused on exercise: patients felt embarrassment at the idea of group exercise<br>• Participants felt able to exercise independently → meant attendance was not necessary
	Barriers regarding expectations of exercised-based CR	• Program-related: Not receiving individual monitoring of cardiovascular fitness and goal settings → expectations unmet<br>• Participants who were not previously active lacked motivation to exercise and social support<br>• Patients perceiving themselves as self-reliant, having resources to support their return to exercise, and being knowledgeable about exercise exertion levels, feeling able to reproduce a "better" exercise programme, having access to gym<br>• Patients perceiving themselves fitter than the exercise intensity levels of exercise-based CR<br>• Comparisons with others perceived to be more suited to exercise-based CR (due to the severity of illness)<br>• Patients considering themselves as being outside age norms → negative attitudes towards exercise-based CR<br>• CR as a reminder of physical losses → decision to not attend<br>• Perception to be already active and conducting alternative PA (walking, gym…) → these activities were less strenuous than CR and more accommodated to physical ability, lifestyle, and social/cultural beliefs → perception that further exercise was not appropriate<br>• CR exercises not individualised
	Illness related barriers	• Perception to manage their condition<br>• Perception that heart attack was "mild"<br>• Perception that medical management helped recovery (CR had no value)

		• Existence of comorbidities
Principal findings and discussion	• Advice given post-event and during CR provided the context for decisions about engagement with exercise-based CR<br>• Prompt contact of individuals diagnosed with ACS and share information<br>• There is a need to clarify the multidisciplinary nature of CR (not primarily an exercise intervention)<br>• For persons immediately post-cardiac event, HCP could benefit from making prompt contact to identify previously active persons and understand their perceptions and confidence<br>• Early communication should highlight the benefits of attending CR such as learning about exercise intensity<br>• Development of a "model of engagement"<br>• Early decision-making was improved by reinforcement of CR benefits, details of aims and objectives, types of CR, and info of supervised environment<br>• Accessing individualised information, for example regarding exercise and age appropriateness<br>• For completing patients, the "fitter self" was at the core of their discussions<br>• Individual assessment of all patients to identify exercise behaviours and support needs → tailored approaches	
Implications for future research	• Need of studies to explore how participants engaging with CR self-regulate exercises independently and in accordance with CR exercise guidelines<br>• How does gender influence the strength of self-perceptions?<br>• To what extent does CR information inflate the perceptions?	
Limitations	• Small number of studies<br>• Mixture of inpatient and outpatient CR	

ACS = acute coronary symptom

<table>
<tr><th colspan="3">Barriers for non-participation and dropout of women in CR (9)</th></tr>
<tr><td>First author</td><td colspan="2">Resurrección, DM</td></tr>
<tr><td>Publication Year</td><td colspan="2">2017</td></tr>
<tr><td>Country</td><td colspan="2">Spain</td></tr>
<tr><td>Study Design</td><td colspan="2">Systematic review</td></tr>
<tr><td>Aims/objectives</td><td colspan="2">To review the literature on barriers perceived by women with CVD affecting their non-participation in and/or dropping out from CR programs</td></tr>
<tr><td>Number of data bases searched</td><td colspan="2">>8</td></tr>
<tr><td>Number of included studies</td><td colspan="2">24</td></tr>
<tr><td>Inclusion criteria</td><td colspan="2">No restrictions</td></tr>
<tr><td>Data synthesis</td><td colspan="2">Using a specified data extraction sheet</td></tr>
<tr><td rowspan="5">Key findings</td><td>Intrapersonal</td><td>• Self-reported health: Feeling too sick, feeling too old, feeling depressed, needing to receive another medical treatment
• Health beliefs: perception that CR is unnecessary, belief that heart attacks cannot be prevented, belief to manage and solve their heart problem by themselves
• Lack of motivation
• Lack of time
• Religious expectations</td></tr>
<tr><td>Interpersonal</td><td>• Lack of family and social support and caretaking responsibilities
• Work conflicts and employment restrictions</td></tr>
<tr><td>Logistical</td><td>• Transport and distance: not having transport, not driving or want to disturb family members
• Personal or community resources</td></tr>
<tr><td>CR program</td><td>• Services offered not adapted to individual needs
• Group format made women feel uncomfortable (talking in groups)
• Exercise component physically too heavy or painful, frightened to exercise
• Inconvenient timing of the program</td></tr>
<tr><td>Health system</td><td>• Lack of referral by physicians, waiting too long to be referred, physician felt it unnecessary to refer them to CR
• Unawareness of CR
• Costs too high, lack of insurance
• Negative experiences with the health system
• Language: CR program not offered in their first language → communication difficulties</td></tr>
<tr><td>Principal findings and discussion</td><td colspan="2">• Women reported multilevel barriers
• Future guidelines should address the barriers to improve adherence
• The heterogeneity in the assessment of barriers should be addressed with a valid instrument</td></tr>
</table>

	• Most findings were reported from the USA
Implications for future research	• More studies from different healthcare contexts are needed<br>• Develop a valid instrument to measure and report barriers
Limitations	• Different study designs included → heterogeneity<br>• No common definition of non-participation and dropout

References

1. **Clark** AM, King-Shier KM, Thompson DR, Spaling MA, Duncan AS, Stone JA, et al. A qualitative systematic review of influences on attendance at cardiac rehabilitation programs after referral. American Heart Journal. 2012;164(6):835-845.e2.
2. **Clark** AM, King-Shier KM, Duncan A, Spaling M, Stone JA, Jaglal S, et al. Factors influencing referral to cardiac rehabilitation and secondary prevention programs: A systematic review. European Journal of Preventive Cardiology. 2013;20(4):692–700.
3. **Ruano-Ravina** A, Pena-Gil C, Abu-Assi E, Raposeiras S, van 't Hof A, Meindersma E, et al. Participation and adherence to cardiac rehabilitation programs. A systematic review. International Journal of Cardiology. 2016;223:436–43.
4. **Resurrección** DM, Moreno-Peral P, Gómez-Herranz M, Rubio-Valera M, Pastor L, Caldas de Almeida JM, et al. Factors associated with non-participation in and dropout from cardiac rehabilitation programmes: a systematic review of prospective cohort studies. European Journal of Cardiovascular Nursing. 2019;18(1):38–47.
5. **Clark** AM, King-Shier KM, Spaling MA, Duncan AS, Stone JA, Jaglal SB, et al. Factors influencing participation in cardiac rehabilitation programmes after referral and initial attendance: qualitative systematic review and meta-synthesis. Clin Rehabil. 2013;27(10):948–59.
6. **Vanzella** L, Oh P, Pakosh M, Ghisi G. Barriers to Cardiac Rehabilitation in Ethnic Minority Groups: A Scoping Review. JOURNAL OF IMMIGRANT AND MINORITY HEALTH. 2021;23(4):824–39.
7. **Supervía** M, Medina-Inojosa JR, Yeung C, Lopez-Jimenez F, Squires RW, Pérez-Terzic CM, et al. Cardiac Rehabilitation for Women: A Systematic Review of Barriers and Solutions. Mayo Clinic Proceedings. 2017;92(4):565–77.
8. **McHale** S, Astin F, Neubeck L, Dawkes S, Hanson CL. A systematic review and thematic synthesis exploring how a previous experience of physical activity influences engagement with cardiac rehabilitation. Eur J Cardiovasc Nurs. 2020;19(1):31–43.
9. **Resurrección** DM, Motrico E, Rigabert A, Rubio-Valera M, Conejo-Cerón S, Pastor L, et al. Barriers for Nonparticipation and Dropout of Women in Cardiac Rehabilitation Programs: A Systematic Review. Journal of Women's Health. 2017;26(8):849–59.
